# Survival Benefit of Three Different Therapies in Postoperative Patients With Advanced Gastric Cancer: A Network Meta-Analysis

**DOI:** 10.3389/fphar.2018.00929

**Published:** 2018-08-22

**Authors:** Dong-Mei Wu, Shan Wang, Xin Wen, Xin-Rui Han, Yong-Jian Wang, Min Shen, Shao-Hua Fan, Zi-Feng Zhang, Juan Zhuang, Qun Shan, Meng-Qiu Li, Bin Hu, Chun-Hui Sun, Jun Lu, Yuan-Lin Zheng

**Affiliations:** ^1^Key Laboratory for Biotechnology on Medicinal Plants of Jiangsu Province, School of Life Science, Jiangsu Normal University, Xuzhou, China; ^2^College of Health Sciences, Jiangsu Normal University, Xuzhou, China

**Keywords:** gastric cancer, network meta-analysis, chemoradiotherapy, overall survival, progression-free survival

## Abstract

**Purpose:** Gastric cancer is mainly treated by gastrectomy, the results of which were unsatisfactory without any adjuvant treatments. This study aimed to examine the performance of radiotherapy, chemotherapy, and chemoradiotherapy after surgery in order to acquire the optimal adjuvant treatment.

**Method:** Embase and PubMed were retrieved to conduct a systematic research. Hazard ratios (HR) of overall survival (OS) and progression-free survival (PFS) as outcomes were calculated by synthesizing direct and indirect evidence to evaluate the efficacy of three treatments against surgery alone. The *P*-score ranking was utilized to rank the therapies. Consistency was assessed by heat plot. Begg's test was performed to evaluate publication bias.

**Results:** A total of 35 randomized controlled studies (RCTs) with 8973 patients were included in our network meta-analysis (NMA). As for efficacy outcomes, OS and PFS of 1, 2, 3, and 5 years, all revealed chemoradiotherapy (CRT) as the best of three adjuvant therapies. Meanwhile, *P*-score ranking results also displayed that CRT was the optimal regimen. Additionally, radiotherapy (RT) and chemotherapy (CT) were two alternative options following CRT since RT performed well in short-term survival while CT could improve the long-term survival.

**Conclusion:** CRT was the most recommended therapy to accompany surgery according to our results. However, no analysis about the safety of these three treatments was mentioned in our study. Further studies including safety outcomes were required to draw a more comprehensive conclusion.

## Introduction

Gastric cancer (GC) is the fourth most common malignant disease and the second most frequent cause of cancer-related deaths worldwide (Duo-Ji et al., 2017). Although the incidence of gastric cancer has declined in the past century, it is still one of the fatal diseases worldwide especially in developing countries (Abe et al., 1988). In 2008, up to 989,600 new gastric cancer cases and 738,000 related cases were reported (Song et al., 2017). Gastric cancer is often diagnosed at an advanced stage because there are no early signs or symptoms. Once the tumor invades over submucosa, it enters into advanced stage, according to Borrmann' classification (Hu et al., 2012). Patients with advanced gastric cancer often suffer from weight loss, abdominal pain, nausea, or vomiting. So far, gastrectomy is still the only curative treatment for advanced gastric cancer (Duo-Ji et al., 2017). However, the results of gastrectomy are often unsatisfactory for patients' high loco-regional recurrence rate (Duo-Ji et al., 2017) and poor survival rate (Desiderio et al., 2017). Most patients with gastric cancer still tend to relapse even in developed regions, and the 5-year overall survival rate stays nearly 40%. To offset the disadvantages of surgery alone, many explorations have been made to find out effective adjuvant therapies, including CT, RT, and CRT.

Radiotherapy (RT) plays an important role in clinic as one of the optional adjuvant treatments for patients with advanced gastric cancer. Radiation can interfere with the growth cycle of cells by damaging DNA replication and lead cancer cells to death. Although promising results on loco-regional recurrence control have been reported, there are many inevitable adverse effects alongside the use of RT due to surgical complications and patient discomfort. No definitive conclusion has been drawn on its effect of survival time (Abe et al., 1988; Calvo et al., 1992). Adjuvant chemotherapy (CT) is another option for gastric cancer patients after surgery and has been investigated on its potential to reduce the recurrence rate and increase the survival rate (Lim et al., 2005). In spite of the fact that postoperative CT can yield better survival results than surgery alone as indicated in some prior meta-analysis (Hermans et al., 1993; Panzini et al., 2002; Norman et al., 2011), several trials still present no statistical difference between postoperative CT and surgery regarding the comparison of OS and PFS (Kulig et al., 2010). The effectiveness of postoperative CT still remains unclear.

To improve the therapeutic efficacy of CT, chemoradiotherapy (CRT) was combined with CT and RT as a more comprehensive therapy (Smalley et al., 2012) and has shown its significant clinical benefit (Bamias et al., 2010). It was reported that postoperative CRT could significantly improve overall survival (OS) and relapse-free survival compared with surgery alone (Yu et al., 2012). In another trial, one-year, two-year, and three-year OS (1-OS, 2-OS, and 3-OS) rates as well as disease-free survival rate were significantly improved with adjuvant CRT (MacDonald et al., 2001). However, because of the use of radiation, CRT is also associated with high toxicity (Bamias et al., 2010).

Till now, a great amount of pair-wise meta-analysis has been conducted to compare either two of the three postoperative adjuvant therapies (CT, RT, CRT), trying to find out the most effective treatment with respect to survivaloutcomes. However, the results of those existing studies seem to be inconsistent. For instance, Soon et al. suggested that postoperative CRT significantly improved OS compared with CT (Soon et al., 2014) while Min et al. and Huang et al. found that there was no significant difference in terms of OS between CRT and CT (Huang et al., 2013; Min et al., 2014). Moreover, a great amount of pair-wise meta-analyses have been conducted to compare either two of the three postoperative adjuvant therapies (CT, RT, and CRT). But the results of comparison among all three therapies have not been integrally evaluated and no explicit conclusion among the relative efficacy of RT, CT, and CRT has been reached. In addition, conventional meta-analysis can only utilize direct evidence while NMA combines both direct and indirect evidence based on clinical trials and is believed to be of high reference value for clinical practice. In the lacking of direct head-to-head evidence between two therapies, NMA can be conducted if both of them have been compared to a same comparator. That is, an indirect estimate of the treatment A over B can be obtained by comparing trials of A vs. C and B vs. C. The estimate of treatment effect obtained from such an analysis is referred to as “indirect evidence”. By NMA we can analysis the effect of more than two kinds of treatments even in the absence of direct head-to-head evidence between two treatments. Thus we extracted all available data to conduct the first NMA comparing these three popular adjuvant therapies which is of great clinical importance. By this means, the effect of the three therapies on prolonging the survival time of patients could be comprehensively explored and recommendations regarding the optimal treatment could be derived from copious trials.

## Materials and methods

### Search strategy

Embase and PubMed were searched for all eligible RCTs. There was no limitation on the date of publication or the date of trials. Key terms used to select eligible studies included “gastric cancer,” “surgery,” “gastrectomy,” “chemotherapy,” “radiotherapy,” “chemoradiotherapy,” and “randomized controlled trials.”

### Selection criteria

In general, one study would be adopted if it satisfied all the following criteria: (1) all the patients were diagnosed with advanced gastric cancer (TNM classification of malignant tumors, 8th edition, 2016; Brierley, 2017); (2) all the treatments were postoperative; (3) the endpoints included either OS or PFS. Studies that belong to any one of categories below would be excluded: (1) studies without enough information for network analysis; (2) duplicate studies; (3) expert opinions, editorials, letters, case reviews, and reports.

### Outcome measurements and data extraction

1-, 2-, 3-, and 5-yrs OS and PFS would be included as outcomes in this NMA. Although adverse events were reported in some of these studies, they would not be included in this NMA due to the missing data in over 75% of included studies. Two investigators participated in the data extraction process independently. Discrepancies were resolved with the intervention of a third investigator who acted as an arbitrator. For each study, basic information including first author, year of publication, country/region, follow-up, group size, completed size, population for different TNM-stage, median age, gender ratio, and treatment was also extracted.

### Statistical analysis

In order to evaluate the relative effectiveness of the three treatments, Bayesian NMA was adopted to integrate the comparison of network. Statistical analysis of HRs with 95% credible interval (95%CrIs) of OS and PFS, which were used to compare the efficacy of different treatments, was conducted by R software (Version 3.2.5). Considering the included studies might differ in population characteristics and the implementation methods of treatments, there would be different effect sizes among different studies. Hence we allowed true effects vary among studies, which rendered the random effects model to be applied in this NMA. The results of our analysis were presented by forest plots. The consistency analysis was exhibited in heat plot, in which the colors reflected the change in inconsistency when detracting one pair of direct comparison show in column. The warm color indicated an increase in consistency cold color illustrated an decrease. In addition, the *P*-score approach was utilized to rank the efficacy of therapies, with higher scores indicating better effectiveness of prolonging survival time. Based on the point estimates and standard errors of the frequentist network meta-analysis estimates under normality assumption, *P*-scores can be computed as unilateral *p*-values in order to measure the mean probability that one regimen is better than the others (Rücker and Schwarzer, 2015).

Furthermore, the Jadad scale (Table S1) was also used to independently assess the quality of the study included in our network meta-analysis.

## Results

### Literature search

After researching, 4,359 studies were retrieved from the electronic database in total, among which 1,748 studies came from PubMed and 2,611 studies were from Embase. None of unpublished studies were identified. After reviewing title and abstract, 1,875 duplicates were removed and 2449 studies were excluded for the lack of sufficient information such as insufficient data and network connections or being found irrelevant in outcomes or contents (Figure S1). Finally, 35 randomized controlled trials (RCTs) published from 1982 to 2015 were adopted in this NMA to conduct a comprehensive comparison among the three postoperative therapies (Douglass and Stablein, 1982; Schlag et al., 1982; Moertel et al., 1984; Nakajima et al., 1984, 1999; Engstrom et al., 1985; Bonfanti, 1988; Jakesz et al., 1988; Coombes et al., 1990; Krook et al., 1991; Grau et al., 1993; Hallissey et al., 1994; Lise et al., 1995; Macdonald et al., 1995; Neri et al., 1996; Tsavaris et al., 1996; Cirera et al., 1999; Bajetta et al., 2002; Skoropad et al., 2002; Nashimoto et al., 2003; Popiela et al., 2004; Bouché et al., 2005; Nitti et al., 2006; De Vita et al., 2007; Sakuramoto et al., 2007; Di Costanzo et al., 2008; Stahl et al., 2009; Bamias et al., 2010; Kulig et al., 2010; Kwon et al., 2010; Kim et al., 2012; Smalley et al., 2012; Yu et al., 2012; Zhu et al., 2012; Park et al., 2015).

### Characteristics and network of included studies

**Figure 1:**
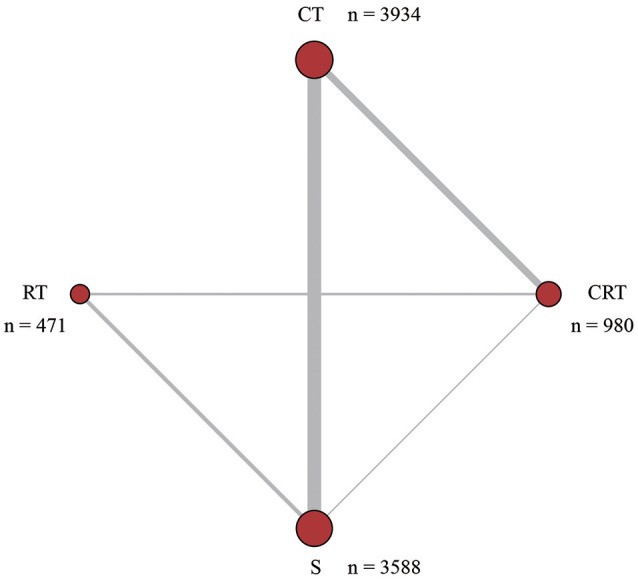
Network structure. The network plots show direct comparison of different treatments, with node size corresponding to the sample size. The number of included studies for specific direct comparison decides the thickness of solid lines. CT, Chemotherapy; RT, Radiotherapy; CRT, Chemoradiotherapy; S, Surgery.

The baseline characteristics of each study were presented in Table 1. As shown in Figure 1, a total of 8,973 patients from various countries or regions were included, among which 980 patients received CRT, 3,934 patients received CT, 471 patients received RT, and 3,588 patients received no adjunctive therapy after surgery. The width of each edge is proportional to the number of RCTs comparing each pair of treatments while the size of each treatment node is proportional to the number of randomized participants (sample size). For the two types of outcome, 15 studies only reported OS and 2 studies only included PFS. In addition, 17 studies included both OS and PFS. The majority of the eligible studies were two-arm trials while one of them was three-arm trials. There are 5,103 patients in stage III/IV, indicating that most patients are in an advanced stage. 4554 patients received D2 lymph node dissection, which also account for a major part of all patients. Male subjects accounted for over 60% of all participants in most of the included trails.

**Table 1:** Main characteristics of included studies.

**References, country**	**Design**	**Follow-up (Months)**	**Type**	**Size/Completed size**	**Men (%)**	**Age**	Stage^*^		**Regimen**	**Outcomes**
							**I/II**	**III/IV**	
(Park et al., 2015), South Korea	RCT	84	CT	228/172	67.00	56 (22–77)	50/86	65/27	Cap and Cis	OS
			CRT	230/188	62.00	56 (28–76)	49/84	71/26	45 Gy RT+ Cap and Cis	
(Zhu et al., 2012), China	RCT	60	CRT	205/186	72.90	56 (38–73)	15/30	96/24	5-FU and L+45 Gy RT	OS
			CT	175/165	76.40	56 (42–75)	20/36	103/27	5-FU and L	
(Smalley et al., 2012), USA	RCT	123.6	CRT	282/182	–	–	175	346/38	5-FU and L+45 Gy RT	OS&PFS
			S	277/266	–	–			–
(Kim et al., 2012), Korea	RCT	117	CRT	46/40	73.90	–	0/0	34/12	5-FU and L+45Gy RT	PFS
			CT	44/41	56.80	–	0/0	33/11	5-FU and L	
(Yu et al., 2012), China	–	36	CRT	34/30	39.29	56	0/3	22/9	5-FU and TF +45Gy RT	OS
			CT	34/34	36.84	57	0/4	20/10	5-FU and TF
(Kwon et al., 2010), Korea	RCT	93	CRT	31/23	67.70	–	0/0	24/7	5-FU and Cis + 45Gy RT and Cap	OS&PFS
			CT	30/22	76.70	–	0/0	27/3	5-FU and Cis	OS
(Kulig et al., 2010), Poland	RCT	65	CT	141/101	71.00	61 (58–67)	20/24	54/43	Et, A and Cis	
			S	154/154	72.00	64 (61–66)	19/32	44/49	–	
(Bamias et al., 2010), Greece	RCT	53.7	CRT	72/49	67.00	63 (32–75)	0/18	47/7	D and Pl + 45Gy RT	OS&PFS
			CT	71/61	73.00	62 (41–79)	3/19	38/10	D and Pl	
(Stahl et al., 2009), German	RCT	65	CRT	60/45	90.00	61	0/0	55/5	Cis, 5-FU, L + 15-17Gy RT	OS&PFS
			CT	59/39	92.00	56	0/0	54/5	Cis, 5-FU, L	
(Di Costanzo et al., 2008), Italy	RCT	73	CT	130/75	61.00	59 (32–73)	19/32	71/7	Cis, Ep, L and 5-FU	OS&PFS
			S	128/–	61.00	59 (18–71)	12/36	75/3	–
(Sakuramoto et al., 2007), Japan	RCT	60	CT	529/–	69.40	63 (27–81)	1/264	224/40	S-1
			S	530/–	69.60	63 (33–80)	0/282	213/35	–	
(De Vita et al., 2007), Italy	RCT	60	CT	112/92	59.00	63 (39–70)	1/38	74/0	Ep, L, 5-FU and Et	OS
			S	113/-	58.00	62 (41–70)	3/35	75/0	–
(Nitti et al., 2006), Belgium	RCT	108	CT	103/76	61.00	56 (29–70)	14/23	61/0	5-FU, A and MTX with L	OS&PFS
			S	103/–	62.00	57 (29–70)	12/27	64/0	–
(Bouché et al., 2005), France	RCT	98	CT	127/79	73.20	60 (32–82)	0/43	65/19	5-FU and Cis	OS&PFS
			S	133/–	69.60	62 (31–83)	0/48	72/10	
(Popiela et al., 2004), Poland	RCT	120	CT	53/–	81.13	58	0/0	38/15	5-FU, A, M	OS
			S	52/–	59.62	60	0/0	42/10	–	
(Nashimoto et al., 2003), Japan	RCT	84	CT	128/80	73.20	58.4 (33–75)	53/67	7/0	M, 5-FU and Cy + oral FU	OS&PFS
			S	123/123	61.80	57.5 (25–75)	53/61	9/0	–
(Skoropad et al., 2002), Russia	RCT	240	RT	51/51	68.63	55 (25–75)	16/16	15/4	20Gy RT	OS
			S	51/51	76.47	54 (36–71)	11/17	18/4	–
(Bajetta et al., 2002), Italy	RCT	66	CT	135/117	60.00	57 (23–70)	65	70	Et, A and Cis + 5-FU and L	OS&PFS
			S	136/128	68.38	57 (31–70)	63	73	–	
(Nakajima et al., 1999), Japan	RCT	72	CT	288/275	60.40	–	97/156	32/0	M, 5-FU + uracil and tegafur	OS
			S	285/281	66.30	–	91/167	30/0	–	
(Cirera et al., 1999), Spain	RCT	76	CT	76/72	68.00	–	1/5	17/53	M+ oral tegafur	OS&PFS
			S	72/72	58.00	–	2/6	22/42	–
(Tsavaris et al., 1996), Greece	RCT	72	CT	42/–	76.19	53 (41–65)	0/19	23/0	5-FU, Ep and M	OS
			S	42/–	59.52	57 (35–66)	0/25	17/0	–	
(Neri et al., 1996), Italy	RCT	36	CT	48/43	68.75	61 (31–70)	0/4	24/20	Ep, L and 5-FU	OS
			S	55/–	70.91	63 (35–73)	1/5	27/22	–
(Macdonald et al., 1995), USA	RCT	180	CT	93/–	63.00	59 (27–75)	17/40	36/0	5-FU, A, M	OS&PFS
			S	100/–	64.00	60 (18–76)	22/42	36/0	–
(Lise et al., 1995), Belgium	RCT	144	CT	155/75	61.00	–	5/63	76/9	5-FU, A, M	OS
			S	159/–	68.00	–	7/63	68/20	–	
(Hallissey et al., 1994), UK	RCT	60	CT	138/58	71.01	63 (58–68)	0/23	71/44	5-FU, A, M	OS
			RT	153/102	64.71	65 (55–69)	0/21	79/53	45Gy RT
			S	145/145	73.10	63 (57–69)	0/27	71/47	–	
(Grau et al., 1993), Spain	RCT	144	CT	68/–	64.71	56 (34–70)	0/31	37/0	M	OS
			S	66/–	66.67	57 (30–70)	0/30	36/0	–
(Krook et al., 1991), USA	RCT	60	CT	61/55	77.00	63 (33–77)	19	42	5-FU, A	OS&PFS
			S	64/–	80.00	62 (38–78)	21	43	–	
(Coombes et al., 1990), USA	RCT	60	CT	133/–	–	–	0/39	92/0	5-FU, A, M	OS&PFS
			S	148/–	–	–	0/41	107/0	–
(Jakesz et al., 1988), Austra	RCT	60	CT	53/–	–	–	0/25	37/25	5-FU, M and Cy	OS
			S	34/–	–	–			–	
(Bonfanti, 1988), Italy	RCT	84	CT	75/67	60.00	–	11/26	38	5-FU, Me-CCNU	OS
			S	69/69	63.77	–	17/23	29	–
(Engstrom et al., 1985), USA	RCT	64	CT	91/–	62.64	–	25	66	5-FU, Me-CCNU	OS&PFS
			S	89/–	70.79	–	23	65	–	
(Nakajima et al., 1984), Japan	RCT	70	CT	154/–	47.40	–	12/21	59/8	M, 5-FU, Cy, F‘	OS
			S	153/–	50.98	–	8/30	47/15	–
(Moertel et al., 1984), USA	RCT	96	CRT	39/–	74.36	–	0/5	12/22	5-FU + RT	PFS
			S	23/–	73.91	56 (41–67)	0/2	8/13	–	
(Schlag et al., 1982), Germany	RCT	36	CT	49/–	61.22	59.8	–	–	5-FU, BCNU	OS&PFS
			S	54/–	61.11	57.6	–	–	–
(Douglass and Stablein, 1982), USA	RCT	60	CT	71/–	70.42	–	–	–	5-FU, Me-CCNU	OS&PFS
			S	71/–	70.42	–	–	–	–

*CT, Chemotherapy; RT, Radiotherapy; CRT, Chemoradiotherapy; S, Surgery; Cap, capecitabine; Cis, cisplatin; FU, fluorouraci; 5-FU, 5-fluorouraci; L, leucovorin; TF, tetrahydrofolic acid; Et, etoposide; A, Adriamycin; D, docetaxel; Pl, platinum; Ep, epirubicin; S-1, Tegafur + 5-FU + Potassium oxonate; MTX, methotrexate; Cy, cytarabine; Me-CCNU, semustine; F‘, ftorafur; BCNU, carmusine; OS,overall survival; PFS, progression-free survival*.

* *The stage of cancer is categorized by TNM classification of malignant tumors, 8th edition*.

### Comparison of treatments

**Figure 2:**
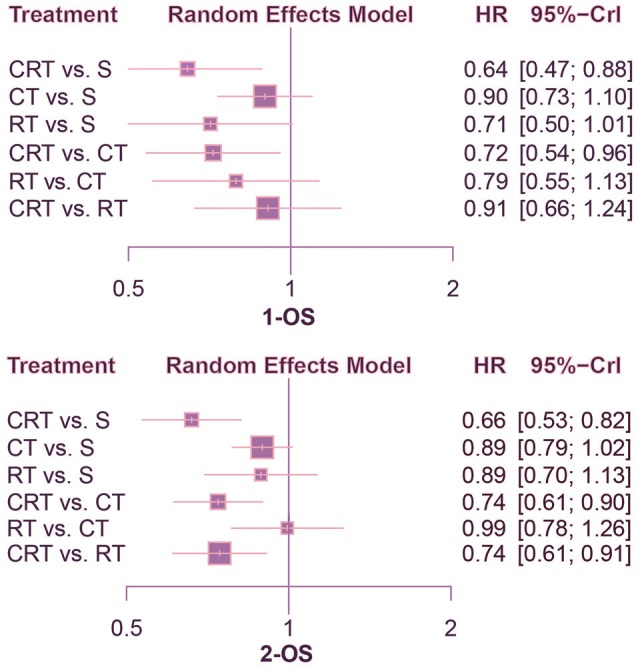
Forest plots for 1- and 2-yr OS. Hazard ratios (HRs) with 95% credible interval (CrIs) indicate the relative efficacy. CT, Chemotherapy; RT, Radiotherapy; CRT, Chemoradiotherapy; S, Surgery; OS, Overall survival.

**Figure 3:**
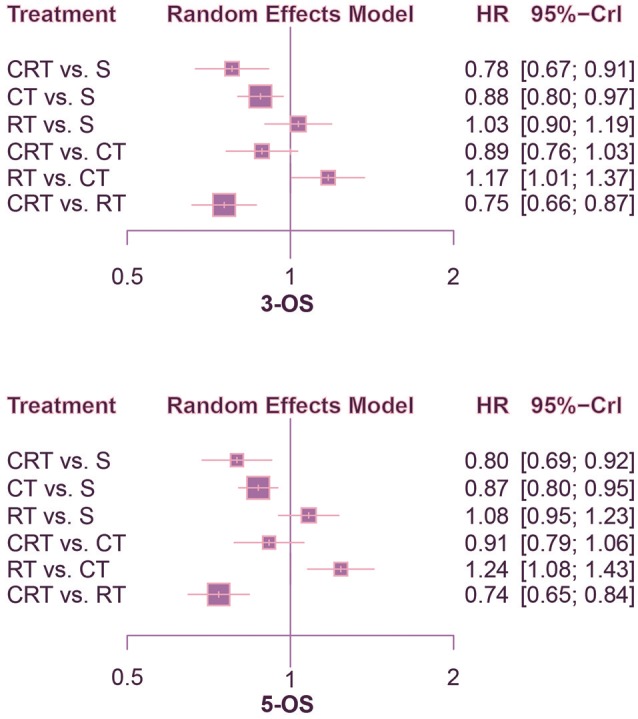
Forest plots for 3- and 5-yr OS. Hazard ratios (HRs) with 95% credible interval (CrIs) indicate the relative efficacy. CT, Chemotherapy; RT, Radiotherapy; CRT, Chemoradiotherapy; S, Surgery; OS, Overall survival.

This NMA was conducted to reveal the relative efficacy on prolonging the survival time of patients. As shown in Figure 2, CRT performed better than both surgery alone and CT in terms of 1-OS (S: HR = 0.64, 95%CrI: 0.47–0.88; CT: HR = 0.72, 95%CrI: 0.54–0.96). With respect to 2-OS, CRT also yielded better outcome than all other treatments (S: HR = 0.66, 95%CrI: 0.53–0.82; CT: HR = 0.74, 95%CrI: 0.61–0.90; RT: HR = 0.74, 95%CrI: 0.61–0.91). The results of 3-OS and 5-OS shown in Figure 3 were similar to the previous results. In terms of 3-OS, CRT and CT revealed better efficacy compared with surgery alone (CRT: HR = 0.78, 95%CrI: 0.67–0.91; CT: HR = 0.88, 95%CrI: 0.80–0.97). They also exhibited a longer survival time when compared with RT (CRT: HR = 0.75, 95%CrI: 0.66–0.87; CT: HR = 0.85, 95%CrI: 0.73–0.99). The similar results existed when comparing 5-OS, with CRT and CT superior to both surgery alone (CRT: HR = 0.80, 95%CrI: 0.69–0.92; CT: HR = 0.87, 95%CrI: 0.80–0.95) and RT (CRT: HR = 0.74, 95%CrI: 0.65–0.84; CT: HR = 0.81, 95%CrI: 0.70–0.93).

**Figure 4:**
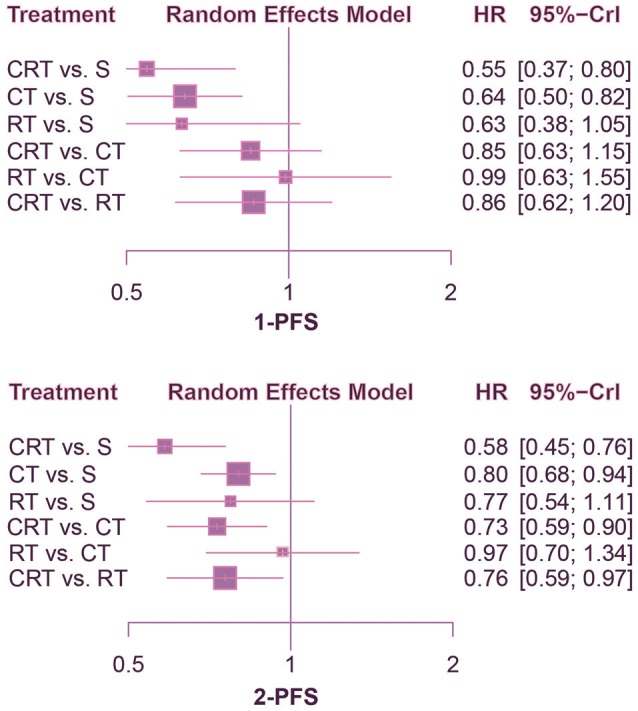
Forest plots for 1- and 2-yr PFS. Hazard ratios (HRs) with 95% credible interval (CrIs) indicate the relative efficacy. CT, Chemotherapy; RT, Radiotherapy; CRT, Chemoradiotherapy; S, Surgery; PFS, Progression-free survival.

**Figure 5:**
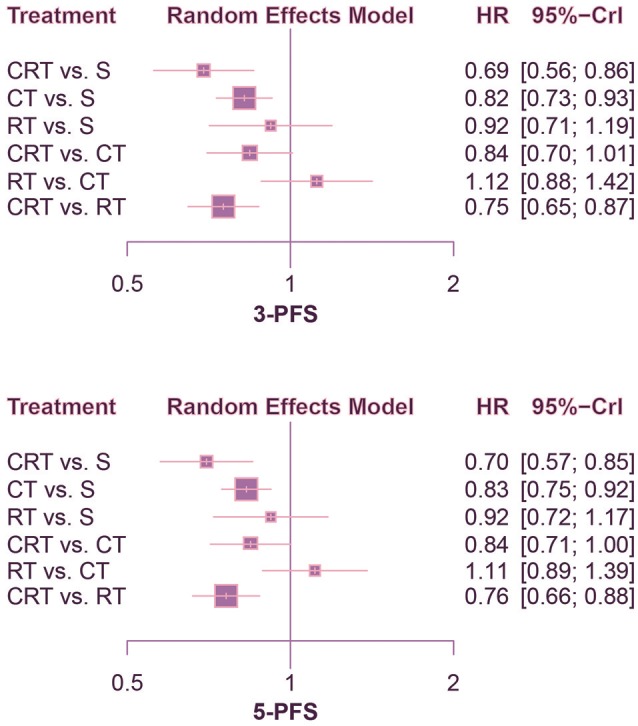
Forest plots for 3- and 5-yr PFS. Hazard ratios (HRs) with 95% credible interval (CrIs) indicate the relative efficacy. CT, Chemotherapy; RT, Radiotherapy; CRT, Chemoradiotherapy; S, Surgery; PFS, Progression-free survival.

According to the comparison of PFS presented in Figures 4, 5, CRT was significantly superior to surgery alone (1-PFS: HR = 0.55, 95%CrI: 0.39–0.80; 2-PFS: HR = 0.58, 95%CrI: 0.45–0.76; 3-PFS: HR = 0.69, 95%CrI: 0.56–0.86; 5-PFS: HR = 0.70, 95%CrI: 0.57–0.85). Moreover, CT showed a significant advantage over surgery as well (1-PFS: HR = 0.64, 95%CrI: 0.50–0.82; 2-PFS: HR = 0.80, 95%CrI: 0.68–0.94; 3-PFS: HR = 0.82, 95%CrI: 0.73–0.93; 5-PFS: HR = 0.83, 95%CrI: 0.75–0.92). Meanwhile there was statistical difference between CRT and CT in terms of 2-PFS (HR = 0.73, 95%CrI: 0.59–0.90). CRT also yielded better outcomes than RT with respect to PFS (2-PFS: HR = 0.76, 95%CrI: 0.59–0.97; 3-PFS: HR = 0.75, 95%CrI: 0.65–0.87; 5-PFS: HR = 0.76, 95%CrI: 0.66–0.88).

### Ranking of treatments

All three adjuvant therapies and surgery alone were sequenced according to *P*-score ranking calculated by software R (version 3.2.5), with the result of which presented in Table 2. According to the *P*-score ranking result, CRT ranked first in all survival terms, which indicated it being the best regimen regarding efficacy of prolonging survival time and progression-free period of patients. RT ranked higher than CT in terms of 1-OS, 1-PFS, and 2-PFS, while the results reversed with respect to 2-OS, 3-OS, 5-OS, 3-PFS, and 5-PFS. Surgery without adjuvant therapies ranked last in all outcome measurements as expected except in 3-OS and 5-OS whereas RT ranked lowest with regard to 3-OS and 5-OS.

**Table 2:** *P*-score ranking.

**Treatment**	**1-OS**	**2-OS**	**3-OS**	**5-OS**	**1-PFS**	**2-PFS**	**3-PFS**	**5-PFS**
CRT	0.905	0.999	0.980	0.961	0.888	0.995	0.989	0.991
CT	0.322	0.479	0.678	0.705	0.540	0.473	0.618	0.615
RT	0.715	0.452	0.115	0.040	0.558	0.505	0.304	0.310
S	0.058	0.070	0.228	0.295	0.013	0.028	0.089	0.084

*CT, Chemotherapy; RT, Radiotherapy; CRT, Chemoradiotherapy; S, Surgery; OS, Overall survival; PFS, Progression-free survival*.

### Consistency test and publication bias

**Figure 6:**
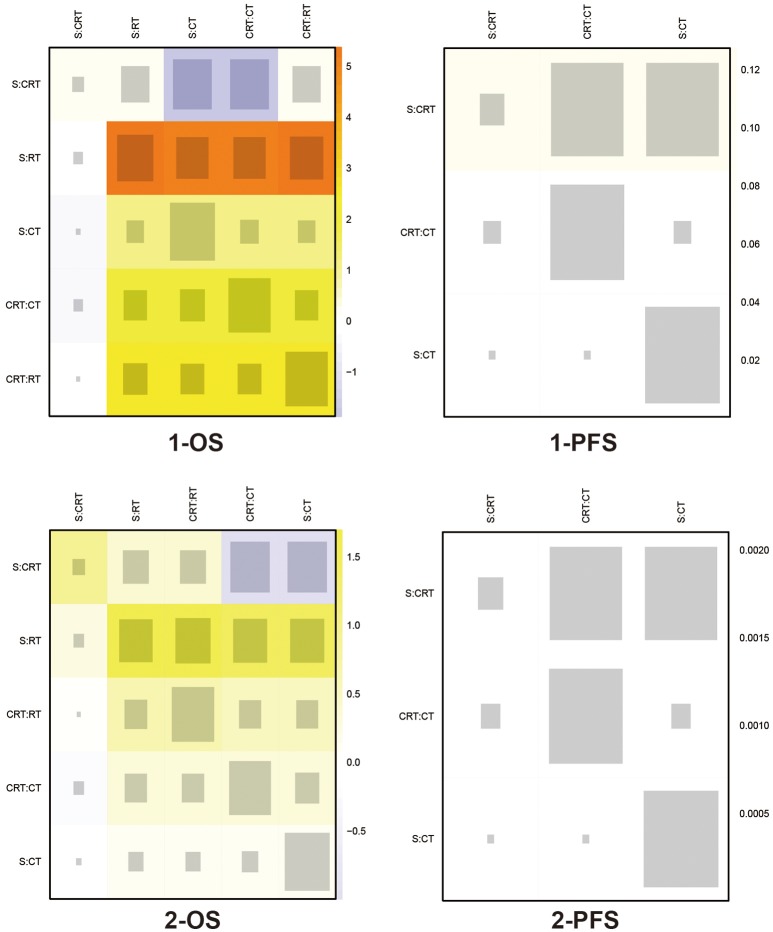
Heat plots for 1- and 2-yr OS, and 1- and 2-yr PFS The size of the gray squares indicates the contribution of the direct evidence (shown in the column) to the network evidence (shown in the row). The colors are associated with the change in inconsistency between direct and indirect evidence (shown in the row). Cold colors indicate a decrease of inconsistency and warm colors indicate an increase. CT, Chemotherapy; RT, Radiotherapy; CRT, Chemoradiotherapy; S, Surgery; OS, Overall survival; PFS, Progression-free survival.

**Figure 7:**
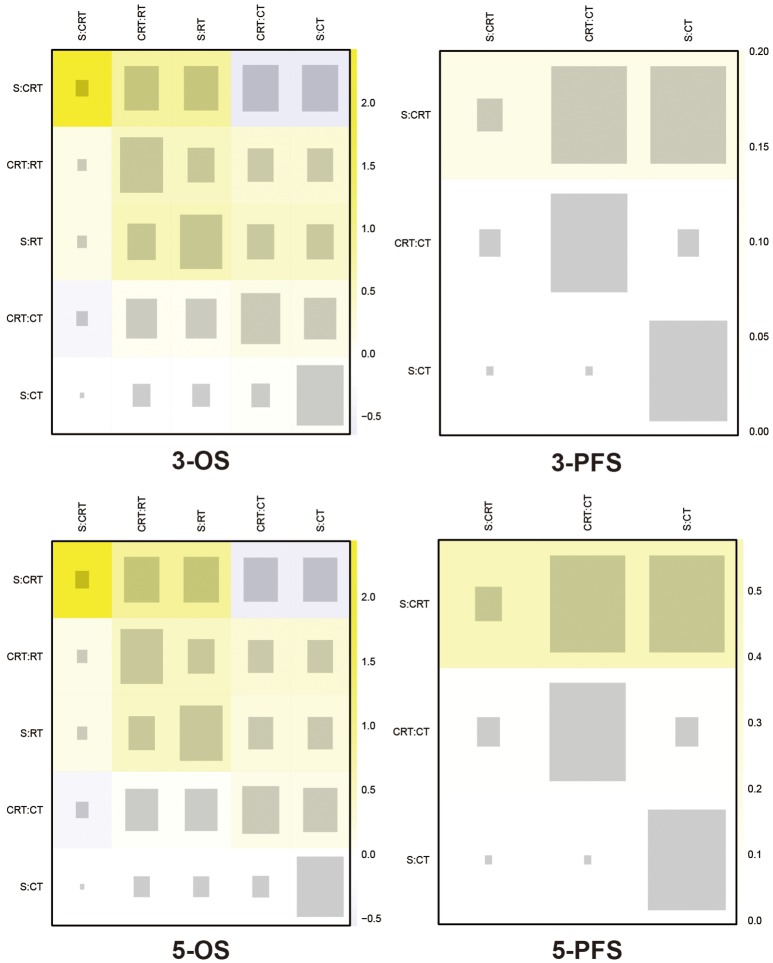
Heat plots for 3- and 5-yr OS, and 3- and 5-yr PFS The size of the gray squares indicates the contribution of the direct evidence (shown in the column) to the network evidence (shown in the row). The colors are associated with the change in inconsistency between direct and indirect evidence (shown in the row). Cold colors indicate a decrease of inconsistency and warm colors indicate an increase. CT, Chemotherapy; RT, Radiotherapy; CRT, Chemoradiotherapy; S, Surgery; OS, Overall survival; PFS, Progression-free survival.

The included trials demonstrated to be of high quality according to Jadad scale we performed in Table S1. There is no evidence of inconsistency among most comparisons as shown in heat plots (Figures 6, 7), which contributed to the reliability of this NMA. However, the comparison in 1-OS should be noticed due to its high possibility of inconsistency. In terms of publication bias, the results of Begg's test (Figure S2) shows that basically no small study effects exist in our NMA.

## Discussion

A total of 35 RCTs with 8,973 patients were included in our NMA. As for efficacy outcomes, which included OS and PFS of 1, 2, 3, and 5 years, all revealed that the excellent performance of CRT as the best of three adjuvant therapies. Meanwhile, *P*-score ranking results also displayed that CRT was the optimal regimen. The optimal status of CRT has been confirmed by prior meta-analyses as mentioned ahead. For instance, papers by Zhou et al., Soon et al., and Dai et al. all suggested a survival benefit of CRT over other adjuvant therapies (Soon et al., 2014; Dai et al., 2015; Zhou et al., 2016). Additionally, RT and CT were two alternative options following CRT since RT performed well in short-term survival while CT could improve the long-term survival.

In this NMA, the exclusion criteria along with the amount of studies with different years of publication guaranteed the reliability of research sources. Although there existed some discrepancy regarding the background of patients and different chemicals utilized in the treatments that may lead to inter-study heterogeneity, we tried to reduce it by restricting the study design and outcome types. Yet subgroup analysis turned out to be impractical for us due to the fact that a large portion of the included studies did not report surgery types while the great majority of those reported surgery types contained not only one kind of surgery. Thus further study could be carried out on the basis of more specific subgroup analysis.

A total of eight outcomes were covered and all the outcomes were measured by HRs with 95%CrIs. According to the forest plots, all the treatments except RT performed better than surgery alone in most outcomes including 1-OS, 3-OS, 5-OS, 1-PFS, 2-PFS, 3-PFS, and 5-PFS, which indicated the absolute efficacy of CT and CRT. However, there was no statistical significance between RT and surgery in any outcomes meanwhile the *P*-score ranking revealed that RT was worse than surgery in long-term measurements. The undesirable performance of RT may be caused by its adverse effects or the insufficiency of trials conducting RT in this NMA. In the meta-analysis carried out by Li et al. (2014), it was found that, in patients with resectable gastric cancer, preoperative RT could improve OS while postoperative RT couldn't. The efficacy variation between preoperative and postoperative RT treatments indicated that further study should be conducted to adequately investigate the value of RT. On the other hand, it turned out that CRT was the most effective treatment because of its great potential to prolong survival time of patients with advanced gastric cancer. CT and RT were alternative options with the former one improving long-term such as 3-year survival and 5-year survival while the latter doing well in short-term survival, such as 1- and 2-year survival. Actually, the effect of CT has been confirmed in previous studies. Notably, the curative effect varies with the corresponding chemotherapeutics. For example, a study conducted by (Di Costanzo et al., 2008; Zhu X. et al., 2016) reported that postoperative chemotherapy based on fluorouracil had little effect on improving long-term survival rate of patients (5-OS: HR = 1.08, 95%CrI = 0.68–1.72) compared with surgery alone, yet another study conducted by (Sakuramoto et al., 2007; Zhu L. et al., 2016) suggested that the use of S-1 in postoperative chemotherapy could significantly benefit long-term survival (5-OS: HR = 0.73, 95%CrI = 0.56–0.94). Different drug combinations may lead to diverse directions thus specific combinations of drugs used in postoperative chemotherapy should be further identified and evaluated. Meanwhile, the safety of different CT regimens is still not clear. Although some studies tried to figure out safety profile, there was still no conclusion (Zhu L. et al., 2016). In addition to CT, the performance of RT should also be further examined. First of all, the sample size of patients treated with RT is relatively small, indicating the insufficiency of evidence supporting NMA results of RT effect. Secondly, most included studies involving RT only demonstrated the comparison of RT and surgery alone, which to some extent made direct comparison between RT and CRT or CT inadequate. Moreover, the implementation of RT can cause a lot of adverse effects on patients, which may dilute its positive impact on survival (Badiani et al., 2015).

As for the tolerability of these therapies, according to current studies, CRT and CT was indicated to show no significant differences in terms of toxicity when treating gastric cancer patients (Li et al., 2014) while CRT has been proved to be well-tolerated after D2 resection (Zhu et al., 2012; Park et al., 2015). However, another study pointed out that approximately 17% patients stopped treatment because of toxicity when received CRT (Smalley et al., 2012), making it more complicated to draw the conclusion toward the safety of CRT and CT. Similar inconsistency existed regarding RT. It was believed having significant toxicity (Bamias et al., 2010) but was shown to be safe when treating patients (Skoropad et al., 2002).

Despite being conducted as scrupulously as possible, this NMA has several limitations.

Firstly, the characteristics of the included studies confined the quality of our analysis. For instance, the most obvious flaw is that the endpoint measuring the level of safety was not covered. In fact, toxicity is an important outcome of adjuvant therapies because patients tend to report adverse events and discomfort as the symptom of cancers after exposure to drugs or radiation. Some of the included studies did report adverse events, such as leucopenia, anemia, fatigue, and diarrhea. However, due to the absence of data in most trials (over 75%), adverse effects could not be assessed in this NMA, which is the reason why no analysis concerning safety was conducted. Besides, the tolerability to the therapies varies among various subpopulations. To be specific, old subjects might be more vulnerable; various races could response differently. Yet due to a lack of sufficient data on population characteristics, a stratified analysis was not conducted in our NMA.

Secondly, blinding methods were different among all included RCTs, which increased the heterogeneity of this study to some extent. However, since only a limited number of trials investigating RT were made, the reason of which may lie in its strong adverse effects, exclusion of this article may also lead to unreliable results. Similarly, the high intensity of inconsistency exists in the comparison of 1-OS between RT and other treatments. Also, the regimen of chemotherapy and the dose of radiotherapy or CRT are different in each study. This difference would have an impact on conclusions in this study, yet according to the final rank, CRT still had an obvious advantage over other treatments.

Thirdly, despite the fact that most included studies were conducted before 2012, the time span from 1982 to 2015 might still be excessive big to some extent. Such a time span might undermine the forwardness of our results. Also, other medical factors such as the development of treatments involved and therapeutic environment might have varied considerably since 1980s. The standards of gastrectomy plus lymphadenectomy in different time are variable. Thus the degree of heterogeneity of our NMA could be increased due to the wide time range.

In conclusion, CRT is the most recommended adjuvant therapy for people with advanced gastric cancer because of its advantage in prolonging long-term survival rate according to the NMA results. Whereas due to the absence of data concerning adverse events of this therapy, its safety assessment still remains unclear, which requires more studies recording adverse events for a more comprehensive analysis.

## Author contributions

D-MW, SW, XW, X-RH, and Y-JW made substantial contribution to the conception and design of the work. MS, S-HF, Z-FZ, JZ, QS, M-QL, BH, and C-HS dealt with the analysis and interpretation of the data. D-MW and SW drafted the manuscript. JL and Y-LZ revised the work critically for important intellectual content and they two were also responsible for the collection of grants. All authors gave final approval of the work.

### Conflict of interest statement

The authors declare that the research was conducted in the absence of any commercial or financial relationships that could be construed as a potential conflict of interest.

## Supplementary material

Figure S1 Flow chart of literature selection.

Figure S2 Results of Begg's test for 1-OS, 2-OS, 3-OS, 5-OS, 1-PFS, 2-PFS, 3-PFS, and 5-PF.

Table S1 Jadad scale for 35 included studies.

## References

[B1] Abe M. Takahashi M. Ono K. Tobe T. Inamoto T. (1988). Japan gastric trials in intraoperative radiation therapy. Int. J. Radiat. Oncol. Biol. Phys. 15, 1431–1433. 10.1016/0360-3016(88)90239-8 3198440

[B2] Badiani B. Maratea D. Messori A. (2015). Second-line treatments for advanced gastric cancer: interpreting outcomes by network meta-analysis. World J. Clin. Oncol. 6, 73–79. 10.5306/wjco.v6.i4.73 26266104 PMC4530381

[B3] Bajetta E. Buzzoni R. Mariani L. Beretta E. Bozzetti F. Bordogna G.. (2002). Adjuvant chemotherapy in gastric cancer: 5-year results of a randomised study by the italian trials in medical oncology (ITMO) group. Ann. Oncol. 13, 299–307. 10.1093/annonc/mdf040 11886009

[B4] Bamias A. Karina M. Papakostas P. Kostopoulos I. Bobos M. Vourli G. (2010). A randomized phase III study of adjuvant platinum/docetaxel chemotherapy with or without radiation therapy in patients with gastric cancer. Cancer Chemother. Pharmacol. 65, 1009–1021. 10.1007/s00280-010-1256-6 20130877

[B5] Bonfanti G. (1988). Adjuvant treatments following curative resection for gastric cancer. Br. J. Surg. 75, 1100–1104. 10.1002/bjs.1800751117 2905188

[B6] Bouché O. Ychou M. Burtin P. Bedenne L. Ducreux M. Lebreton G.. (2005). Adjuvant chemotherapy with 5-fluorouracil and cisplatin compared with surgery alone for gastric cancer: 7-year results of the FFCD randomized phase III trial (8801). Ann. Oncol. 16, 1488–1497. 10.1093/annonc/mdi270 15939717

[B7] Brierley J. D. (2017). TNM Classification of Malignant Tumours. Hoboken, NJ: John Wiley & Sons.

[B8] Calvo F. A. Aristu J. J. Azinovic I. Abuchaibe O. Escude L. Martinez R.. (1992). Intraoperative and external radiotherapy in resected gastric cancer: updated report of a phase II trial. Int. J. Radiat. Oncol. Biol. Phys. 24, 729–736. 10.1016/0360-3016(92)90721-S 1429097

[B9] Cirera L. Balil A. Batiste-Alentorn E. Tusquets I. Cardona T. Arcusa A.. (1999). Randomized clinical trial of adjuvant mitomycin plus tegafur in patients with resected stage III gastric cancer. J. Clin. Oncol. 17, 3810–3815. 10.1200/JCO.1999.17.12.3810 10577853

[B10] Coombes R. C. Schein P. S. Chilvers C. E. D. Wils J. Beretta G. Bliss J. M.. (1990). A randomized trial comparing adjuvant fluorouracil, doxorubicin, and mitomycin with no treatment in operable gastric cancer. J. Clin. Oncol. 8, 1362–1369. 10.1200/JCO.1990.8.8.1362 2199622

[B11] Dai Q. Jiang L. Lin R. J. Wei K. K. Gan L. L. Deng C. H.. (2015). Adjuvant chemoradiotherapy versus chemotherapy for gastric cancer: a meta-analysis of randomized controlled trials. J. Surg. Oncol. 111, 277–284. 10.1002/jso.23795 25273525

[B12] De Vita F. Giuliani F. Orditura M. Maiello E. Galizia G. Di Martino N.. (2007). Adjuvant chemotherapy with epirubicin, leucovorin, 5-fluorouracil and etoposide regimen in resected gastric cancer patients: a randomized phase III trial by the Gruppo Oncologico Italia Meridionale (GOIM 9602 Study). Ann. Oncol. 18, 1354–1358. 10.1093/annonc/mdm128 17525087

[B13] Desiderio J. Chao J. Melstrom L. Warner S. Tozzi F. Fong Y.. (2017). The 30-year experience-A meta-analysis of randomised and high-quality non-randomised studies of hyperthermic intraperitoneal chemotherapy in the treatment of gastric cancer. Eur. J. Cancer 79, 1–14. 10.1016/j.ejca.2017.03.030 28456089 PMC5568419

[B14] Di Costanzo F. Gasperoni S. Manzione L. Bisagni G. Labianca R. Bravi S.. (2008). Adjuvant chemotherapy in completely resected gastric cancer: a randomized phase III trial conducted by GOIRC. J. Natl. Cancer Inst. 100, 388–398. 10.1093/jnci/djn054 18334706

[B15] Douglass H. O. Jr. Stablein D. M. (1982). Controlled trial of adjuvant chemotherapy following curative resection for gastric cancer. Cancer 49, 1116–1122. 7037156 10.1002/1097-0142(19820315)49:6<1116::aid-cncr2820490609>3.0.co;2-u

[B16] Duo-Ji M. M. Ci-Ren B. S. Long Z. W. Zhang X. H. Luo D. L. (2017). Short-term efficacy of different chemotherapy regimens in the treatment of advanced gastric cancer: a network meta-analysis. Oncotarget 8, 37896–37911. 10.18632/oncotarget.14664 28099947 PMC5514960

[B17] Engstrom P. F. Lavin P. T. Douglass H. O. Jr. Brunner K. W. (1985). Postoperative adjuvant 5-fluorouracil plus methyl-CCNU therapy for gastric cancer patients. eastern cooperative oncology group study (EST 3275). Cancer 55, 1868–1873. 3884131 10.1002/1097-0142(19850501)55:9<1868::aid-cncr2820550904>3.0.co;2-b

[B18] Grau J. J. Estapé J. Alcobendas F. Pera C. Daniels M. Teres J. (1993). Positive results of adjuvant mitomycin-C in resected gastric cancer: a randomised trial on 134 patients. Eur. J. Cancer 29A, 340–342. 10.1016/0959-8049(93)90381-O 8398330

[B19] Hallissey M. T. Dunn J. A. Ward L. C. Allum W. H. (1994). The second british stomach cancer group trial of adjuvant radiotherapy or chemotherapy in resectable gastric cancer: five-year follow-up. Lancet 343, 1309–1312. 10.1016/S0140-6736(94)92464-3 7910321

[B20] Hermans J. Bonenkamp J. J. Boon M. C. Bunt A. M. Ohyama S. Sasako M.. (1993). Adjuvant therapy after curative resection for gastric cancer: meta-analysis of randomized trials. J. Clin. Oncol. 11, 1441–1447. 10.1200/JCO.1993.11.8.1441 8336183

[B21] Hu B. El Hajj N. Sittler S. Lammert N. Barnes R. Meloni-Ehrig A. (2012). Gastric cancer: classification, histology and application of molecular pathology. J. Gastrointest. Oncol. 3, 251–261. 10.3978/j.issn.2078-6891.2012.021 22943016 PMC3418539

[B22] Huang Y. Y. Yang Q. Zhou S. W. Wei Y. Chen Y. X. Xie D. R.. (2013). Postoperative chemoradiotherapy versus postoperative chemotherapy for completely resected gastric cancer with D2 Lymphadenectomy: a meta-analysis. PLoS ONE 8:e68939. 10.1371/journal.pone.0068939 23874819 PMC3715514

[B23] Jakesz R. Dittrich C. Funovics J. Hofbauer F. Rainer H. Reiner G.. (1988). The effect of adjuvant chemotherapy in gastric carcinoma is dependent on tumor histology: 5-year results of a prospective randomized trial. Recent Results Cancer Res. 110, 44–51. 10.1007/978-3-642-83293-2_6 3043596

[B24] Kim T. H. Park S. R. Ryu K. W. Kim Y. W. Bae J. M. Lee J. H.. (2012). Phase 3 trial of postoperative chemotherapy alone versus chemoradiation therapy in stage III-IV gastric cancer treated with R0 gastrectomy and D2 lymph node dissection. Int. J. Radiat. Oncol. Biol. Phys. 84:e585–592. 10.1016/j.ijrobp.2012.07.2378 22975616

[B25] Krook J. E. O'Connell M. J. Wieand H. S. Beart R. W. Jr. Leigh J. E. Kugler J. W.. (1991). A prospective, randomized evaluation of intensive-course 5-fluorouracil plus doxorubicin as surgical adjuvant chemotherapy for resected gastric cancer. Cancer 67, 2454-2458. 2015545 10.1002/1097-0142(19910515)67:10<2454::aid-cncr2820671010>3.0.co;2-2

[B26] Kulig J. Kolodziejczyk P. Sierzega M. Bobrzynski L. Jedrys J. Popiela T.. (2010). Adjuvant chemotherapy with etoposide, adriamycin and cisplatin compared with surgery alone in the treatment of gastric cancer: a phase III randomized, multicenter, clinical trial. Oncology 78, 54–61. 10.1159/000292360 20215786

[B27] Kwon H. C. Kim M. C. Kim K. H. Jang J. S. Oh S. Y. Kim S. H.. (2010). Adjuvant chemoradiation versus chemotherapy in completely resected advanced gastric cancer with D2 nodal dissection. Asia Pac. J. Clin. Oncol. 6, 278–285. 10.1111/j.1743-7563.2010.01331.x 21114777

[B28] Li L. L. Xie C. Y. Su H. F. (2014). Benefit of radiotherapy on survival in resectable gastric carcinoma: a meta-analysis. Tumour Biol. 35, 4957–4966. 10.1007/s13277-014-1653-2 24500665

[B29] Lim L. Michael M. Mann G. B. Leong T. (2005). Adjuvant therapy in gastric cancer. J. Clin. Oncol. 23, 6220–6232. 10.1200/JCO.2005.11.593 16135489

[B30] Lise M. Nitti D. Marchet A. Sahmoud T. Buyse M. Duez N.. (1995). Final results of a phase III clinical trial of adjuvant chemotherapy with the modified fluorouracil, doxorubicin, and mitomycin regimen in resectable gastric cancer. J. Clin. Oncol. 13, 2757–2763. 10.1200/JCO.1995.13.11.2757 7595735

[B31] Macdonald J. S. Fleming T. R. Peterson R. F. Berenberg J. L. Mcclure S. Chapman R. A.. (1995). Adjuvant chemotherapy with 5-FU, adriamycin, and mitomycin-C (FAM) versus surgery alone for patients with locally advanced gastric adenocarcinoma: a southwest oncology group study. Ann. Surg. Oncol. 2, 488–494. 10.1007/BF02307081 8591078

[B32] MacDonald J. S. Smalley S. R. Benedetti J. Hundahl S. A. Estes N. C. Stemmermann G. N. (2001). Chemoradiotherapy after surgery compared with surgery alone for adenocarcinoma of the stomach or gastroesophageal junction. N. Engl. J. Med. 345, 725–730. 10.1056/NEJMoa010187 11547741

[B33] Min C. Bangalore S. Jhawar S. Guo Y. Nicholson J. Formenti S. C.. (2014). Chemoradiation therapy versus chemotherapy alone for gastric cancer after R0 surgical resection: a meta-analysis of randomized trials. Oncology 86, 79–85. 10.1159/000354641 24435019

[B34] Moertel C. G. Childs D. S. O'fallon J. R. (1984). Combined 5-fluorouracil and radiation therapy as a surgical adjuvant for poor prognosis gastric carcinoma. J. Clin. Oncol. 2, 1249–1254. 10.1200/JCO.1984.2.11.1249 6491703

[B35] Nakajima T. Nashimoto A. Kitamura M. Kito T. Iwanaga T. Okabayashi K.. (1999). Adjuvant mitomycin and fluorouracil followed by oral uracil plus tegafur in serosa-negative gastric cancer: a randomised trial. Lancet 354, 273–277. 10.1016/S0140-6736(99)01048-X 10440302

[B36] Nakajima T. Takahashi T. Takagi K. Kuno K. Kajitani T. (1984). Comparison of 5-fluorouracil with ftorafur in adjuvant chemotherapies with combined inductive and maintenance therapies for gastric cancer. J. Clin. Oncol. 2, 1366–1371. 10.1200/JCO.1984.2.12.1366 6439835

[B37] Nashimoto A. Nakajima T. Furukawa H. Kitamura M. Kinoshita T. Yamamura Y.. (2003). Randomized trial of adjuvant chemotherapy with mitomycin, fluorouracil, and cytosine arabinoside followed by oral fluorouracil in serosa-negative gastric cancer: Japan clinical oncology group 9206-1. J. Clin. Oncol. 21, 2282–2287. 10.1200/JCO.2003.06.103 12805327

[B38] Neri B. de Leonardis V. Romano S. Andreoli F. Pernice L. M. Bruno L.. (1996). Adjuvant chemotherapy after gastric resection in node-positive cancer patients: a multicentre randomised study. Br. J. Cancer 73, 549–552. 10.1038/bjc.1996.95 8595173 PMC2074461

[B39] Nitti D. Wils J. Dos Santos J. G. Fountzilas G. Conte P. F. Sava C. (2006). Randomized phase III trials of adjuvant FAMTX or FEMTX compared with surgery alone in resected gastric cancer. A combined analysis of the EORTC GI Group and the ICCG. Ann. Oncol. 17, 262–269. 10.1093/annonc/mdj077 16293676

[B40] Norman G. Rice S. Spackman E. Stirk L. Danso-Appiah A. Suh D. (2011). Trastuzumab for the treatment of HER2-positive metastatic adenocarcinoma of the stomach or gastro-oesophageal junction. Health Technol. Assess. 15(Suppl. 1), 33–42. 10.3310/hta15suppl1/04 21609651

[B41] Panzini I. Gianni L. Fattori P. P. Tassinari D. Imola M. Fabbri P.. (2002). Adjuvant chemotherapy in gastric cancer: a meta-analysis of randomized trials and a comparison with previous meta-analyses. Tumori 88, 21–27. 12004845

[B42] Park S. H. Sohn T. S. Lee J. Lim D. H. Hong M. E. Kim K. M.. (2015). Phase III trial to compare adjuvant chemotherapy with capecitabine and cisplatin versus concurrent chemoradiotherapy in gastric cancer: final report of the adjuvant chemoradiotherapy in stomach tumors trial, including survival and subset analyses. J. Clin. Oncol. 33, 3130–3136. 10.1200/JCO.2014.58.3930 25559811

[B43] Popiela T. Kulig J. Czupryna A. Szczepanik A. M. Zembala M. (2004). Efficiency of adjuvant immunochemotherapy following curative resection in patients with locally advanced gastric cancer. Gastric Cancer 7, 240–245. 10.1007/s10120-004-0299-y 15616772

[B44] Rücker G. Schwarzer G. (2015). Ranking treatments in frequentist network meta-analysis works without resampling methods. BMC Med. Res. Methodol. 15:58 10.1186/s12874-015-0060-8 26227148 PMC4521472

[B45] Sakuramoto S. Sasako M. Yamaguchi T. Kinoshita T. Fujii M. Nashimoto A.. (2007). Adjuvant chemotherapy for gastric cancer with S-1, an oral fluoropyrimidine. N. Engl. J. Med. 357, 1810–1820. 10.1056/NEJMoa072252 17978289

[B46] Schlag P. Schreml W. Gaus W. Herfarth C. Linder M. M. Queisser W.. (1982). Adjuvant 5-fluorouracil and BCNU chemotherapy in gastric cancer: 3-year results. Recent Results Cancer Res. 80, 277–283. 10.1007/978-3-642-81685-7_44 7036292

[B47] Skoropad V. Berdov B. Zagrebin V. (2002). Concentrated preoperative radiotherapy for resectable gastric cancer: 20-years follow-up of a randomized trial. J. Surg. Oncol. 80, 72–78. 10.1002/jso.10102 12173383

[B48] Smalley S. R. Benedetti J. K. Haller D. G. Hundahl S. A. Estes N. C. Ajani J. A.. (2012). Updated analysis of SWOG-directed intergroup study 0116: a phase III trial of adjuvant radiochemotherapy versus observation after curative gastric cancer resection. J. Clin. Oncol. 30, 2327–2333. 10.1200/JCO.2011.36.7136 22585691 PMC4517071

[B49] Song G. M. Liu X. L. Bian W. Wu J. Deng Y. H. Zhang H.. (2017). Systematic review with network meta-analysis: comparative efficacy of different enteral immunonutrition formulas in patients underwent gastrectomy. Oncotarget 8, 23376–23388. 10.18632/oncotarget.15580 28423579 PMC5410311

[B50] Soon Y. Y. Leong C. N. Tey J. C. Tham I. W. Lu J. J. (2014). Postoperative chemo-radiotherapy versus chemotherapy for resected gastric cancer: a systematic review and meta-analysis. J. Med. Imaging Radiat. Oncol. 58, 483–496. 10.1111/1754-9485.12190 24995607

[B51] Stahl M. Walz M. K. Stuschke M. Lehmann N. Meyer H. J. Riera-Knorrenschild J.. (2009). Phase III comparison of preoperative chemotherapy compared with chemoradiotherapy in patients with locally advanced adenocarcinoma of the esophagogastric junction. J. Clin. Oncol. 27, 851–856. 10.1200/JCO.2008.17.0506 19139439

[B52] Tsavaris N. Tentas K. Kosmidis P. Mylonakis N. Sakelaropoulos N. Kosmas C.. (1996). A randomized trial comparing adjuvant fluorouracil, epirubicin, and mitomycin with no treatment in operable gastric cancer. Chemotherapy 42, 220–226. 10.1159/000239446 8983891

[B53] Yu C. Yu R. Zhu W. Song Y. Li T. (2012). Intensity-modulated radiotherapy combined with chemotherapy for the treatment of gastric cancer patients after standard D1/D2 surgery. J. Cancer Res. Clin. Oncol. 138, 255–259. 10.1007/s00432-011-1085-y 22105898 PMC11824713

[B54] Zhou M. L. Kang M. Li G. C. Guo X. M. Zhang Z. (2016). Postoperative chemoradiotherapy versus chemotherapy for R0 resected gastric cancer with D2 lymph node dissection: an up-to-date meta-analysis. World J. Surg. Oncol. 14:209 10.1186/s12957-016-0957-7 27502921 PMC4977857

[B55] Zhu L. Liu J. Ma S. (2016). Fluoropyrimidine-based chemotherapy as first-line treatment for advanced gastric cancer: a bayesian network meta-analysis. Pathol. Oncol. Res. 22, 853–861. 10.1007/s12253-016-0078-1 27236591

[B56] Zhu W. G. Xua D. F. Pu J. Zong C. D. Li T. Tao G. Z.. (2012). A randomized, controlled, multicenter study comparing intensity-modulated radiotherapy plus concurrent chemotherapy with chemotherapy alone in gastric cancer patients with D2 resection. Radiother. Oncol. 104, 361–366. 10.1016/j.radonc.2012.08.024 22985776

[B57] Zhu X. Ko Y. J. Berry S. Shah K. Lee E. Chan K. (2016). A Bayesian network meta-analysis on second-line systemic therapy in advanced gastric cancer. Gastric Cancer 20, 646–654. 10.1007/s10120-016-0656-7 27722826

