# Supplementary figures and images for: Survival Benefit of Three Different Therapies in Postoperative Patients With Advanced Gastric Cancer: A Network Meta-Analysis
Source: Front Pharmacol. 2018 Aug 22;9:929. doi: 10.3389/fphar.2018.00929 (PMC6119769; doi:10.3389/fphar.2018.00929)

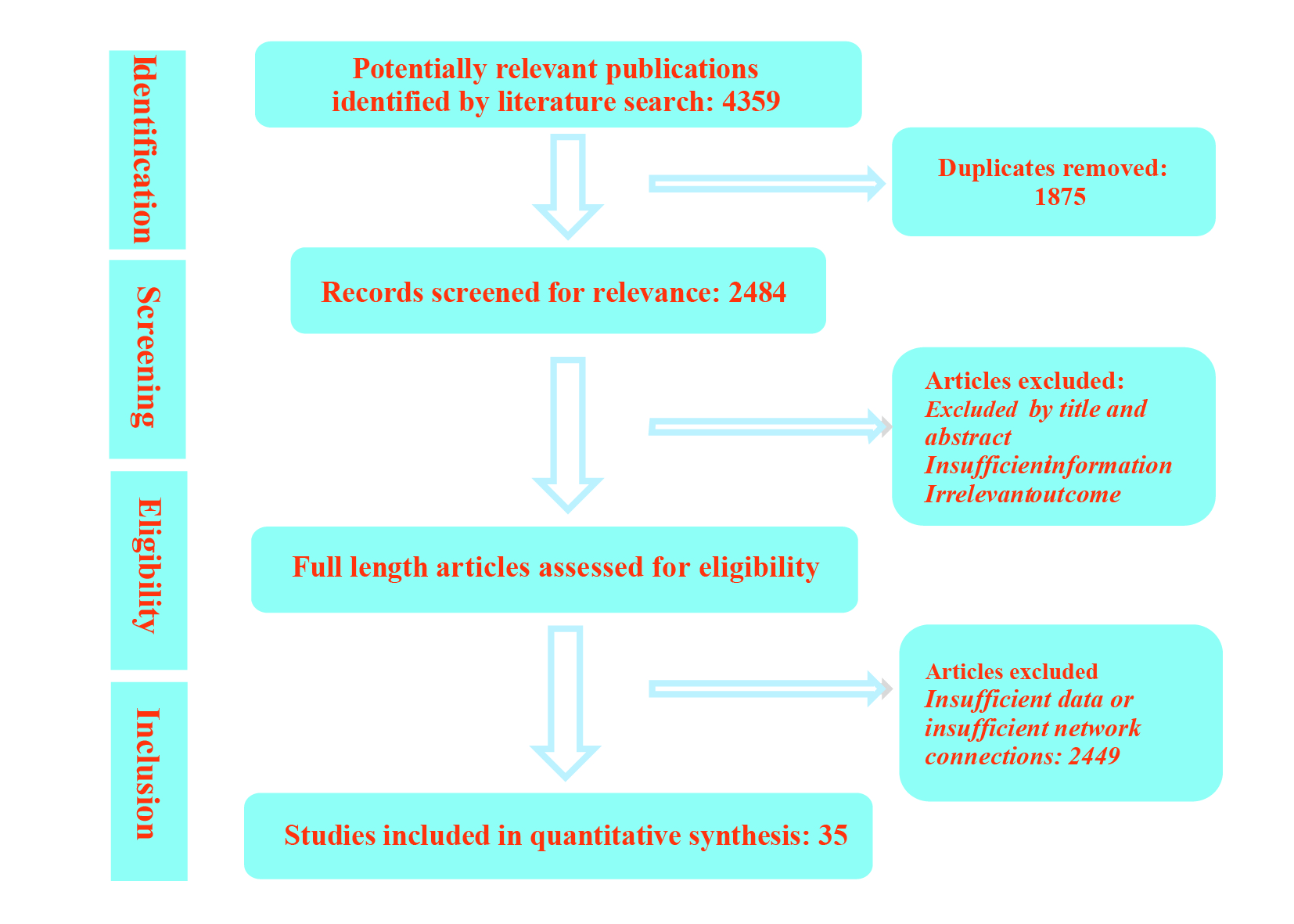

Supplement: Figure S1 — Flow chart of literature selection. [file Image_1.JPEG]

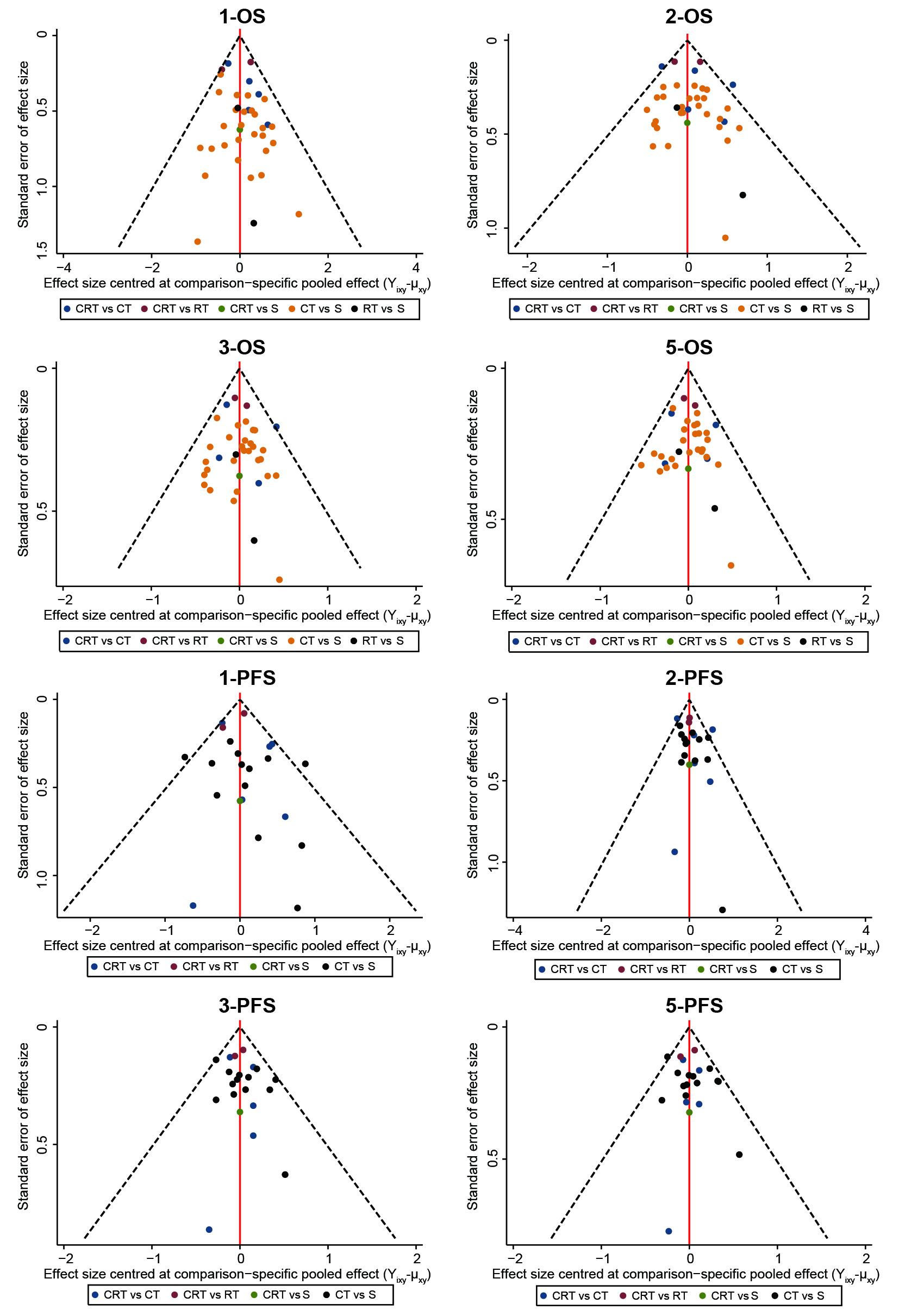

Supplement: Figure S2 — Results of Begg's test for 1-OS, 2-OS, 3-OS, 5-OS, 1-PFS, 2-PFS, 3-PFS, and 5-PF. [file Image_2.JPEG]
