# Supplementary material for: Survival Benefit of Three Different Therapies in Postoperative Patients With Advanced Gastric Cancer: A Network Meta-Analysis
Source: Front Pharmacol. 2018 Aug 22;9:929. doi: 10.3389/fphar.2018.00929 (PMC6119769; doi:10.3389/fphar.2018.00929)
Supplement: Table S1 — Jadad scale for 35 included studies. [file Table_1.docx]

**Table S1. Jadad Scale for 35 Included Studies**

| **Author** | **Blinding** | **Randomization** | **An account of patients** | **High quality** |
| --- | --- | --- | --- | --- |
| Park | 2 | 2 | 0 | √ |
| Zhu | 0 | 2 | 1 | √ |
| Smalley | 0 | 2 | 1 | √ |
| Kim | 0 | 2 | 1 | √ |
| Yu | 0 | 2 | 1 | √ |
| Kwon | 0 | 2 | 1 | √ |
| Kulig | 0 | 2 | 1 | √ |
| Bamias | 2 | 2 | 1 | √ |
| Stahl | 0 | 2 | 1 | √ |
| Di Costanzo | 0 | 2 | 1 | √ |
| Sakuramoto | 0 | 2 | 1 | √ |
| De Vita | 0 | 2 | 1 | √ |
| Nitti | 0 | 2 | 1 | √ |
| Bouche | 0 | 2 | 1 | √ |
| Popiela | 0 | 2 | 1 | √ |
| Nashimoto | 0 | 2 | 1 | √ |
| Skoropad | 0 | 2 | 1 | √ |
| Bajetta | 0 | 2 | 1 | √ |
| Nakajima | 0 | 2 | 1 | √ |
| Cirera | 0 | 2 | 1 | √ |
| Tsavaris | 0 | 2 | 1 | √ |
| Neri | 0 | 2 | 1 | √ |
| Macdonald | 2 | 2 | 0 | √ |
| Lise | 0 | 2 | 1 | √ |
| Hallissey | 0 | 2 | 1 | √ |
| Grau | 0 | 2 | 1 | √ |
| Krook | 0 | 2 | 1 | √ |
| Coombes | 0 | 2 | 1 | √ |
| Jakesz | 0 | 2 | 1 | √ |
| Bonfanti | 0 | 2 | 1 | √ |
| Engstrom | 2 | 2 | 1 | √ |
| Mertel | 2 | 2 | 1 | √ |
| Nakajima | 0 | 2 | 1 | √ |
| Schlag | 0 | 2 | 1 | √ |
| Douglass | 2 | 2 | 1 | √ |

* When total score≥3, the relative RCT will be considered as of high quality.

The questions were as follows: 1. Was the study described as randomized? 2. Was the study described as double blind? 3. Was there a description of withdrawals and dropouts? To receive the corresponding point, an article should describe the number of withdrawals and dropouts, in each of the study groups, and the underlying reasons. Additional points were given if: 1. The method of randomization was described in the paper, and that method was appropriate. (1 extra point in randomization part); 2. The method of blinding was described, and it was appropriate. (1 extra point in blinding part)
